# TI: NLRP3 Inflammasome-Dependent Pyroptosis in CNS Trauma: A Potential Therapeutic Target

**DOI:** 10.3389/fcell.2022.821225

**Published:** 2022-02-02

**Authors:** Conghui Zhou, Jinfeng Zheng, Yunpeng Fan, Junsong Wu

**Affiliations:** ^1^ The First Affiliated Hospital of Zhejiang University, School of Medicine, Zhejiang University, Hangzhou, China; ^2^ Department of Orthopaedics of the First Affiliated Hospital, School of Medicine, Zhejiang University, Hangzhou, China

**Keywords:** NLRP3, pyroptosis, spinal cord injury, traumatic brain injury, CNS trauma

## Abstract

Central nervous system (CNS) trauma, including traumatic brain injury (TBI) and traumatic spinal cord injury (SCI), is characterized by high morbidity, disability, and mortality. TBI and SCI have similar pathophysiological mechanisms and are often accompanied by serious inflammatory responses. Pyroptosis, an inflammation-dependent programmed cell death, is becoming a major problem in CNS post-traumatic injury. Notably, the pyrin domain containing 3 (NLRP3) inflammasome is a key protein in the pyroptosis signaling pathway. Therefore, underlying mechanism of the NLRP3 inflammasome in the development of CNS trauma has attracted much attention. In this review, we briefly summarize the molecular mechanisms of NLRP3 inflammasome in pyroptosis signaling pathway, including its prime and activation. Moreover, the dynamic expression pattern, and roles of the NLRP3 inflammasome in CNS post-traumatic injury are summarized. The therapeutic applications of NLRP3 inflammasome activation inhibitors are also discussed.

## 1 Introduction

Central nervous system (CNS) traumatic diseases, including spinal cord injury (SCI) and traumatic brain injury (TBI), are characterized by a high disability rate and various complications, and some hypotheses propose that high disability and mortality rates occur because of secondary injury development. Surgical treatment remains an effective clinical method. Olfactory ensheathing cell transplantation, hematopoietic stem cell transplantation, hematopoietic progenitor cell transplantation, novel cell transdifferentiation techniques, or combined with scaffold therapy have presented new possibilities for nerve repair (Bryukhovetskiy, 2018; Ma et al., 2019; Kuang et al., 2021). Additionally, SCI and TBI share common pathological features, and their complex pathophysiological processes can be classified as primary and secondary injuries. Primary injury is caused by direct external force to the spinal cord or brain, and the damage caused at this stage is irreversible. Delayed secondary injury cascade involves several complex pathophysiological events, including glutamate excitotoxicity, oxidative stress, and inflammatory response (Toklu et al., 2009; Zendedel et al., 2016). Glutamate over-release stimulates glutamate receptors, leading to persistent excitatory toxicity, cell dysfunction, and death after SCI and TBI, and Na^+^-dependent glutamate transporters GLT-1 and GLAST actively transport excess glutamate, although downregulated after injury (Yi and Hazell, 2006; Fernandez-Lopez et al., 2014). Furthermore, glutamate-induced stimulation is mediated by Na^+^/K^+^-ATPase in addition to Na^+^/glutamate cotransporter (Pellerin and Magistretti, 1994). Na^+^/K^+^-ATPase dysfunction is closely related to SCI and TBI and can indirectly reflect the severity of the injury. The Na^+^/K^+^-ATPase pump, in particular, is highly sensitive to oxidative stress (Lima et al., 2008; Boon et al., 2012).

As a response of the innate immune system in the CNS, neuroinflammation plays a key role in the second stage of TBI and SCI, of which the most important step is the activation of the various inflammasome complexes (Zhou et al., 2016). Additionally, the occurrence of neuroinflammation not only represents damage to the brain and spinal cord, especially the activation of macrophages and microglia, but is also is known to be an essential protective mechanism (Mortezaee et al., 2018). However, excessive neuroinflammation tends to hinder repair and regeneration, and the NOD-like receptor family, pyrin domain containing 3 (NLRP3) inflammasome-mediated neuroinflammation appears to be responsible for the unfavorable outcome in CNS injury. Activation of the NLRP3 inflammasome is the key to pyroptosis and the trigger of the inflammatory response, which plays an important role in the pathogenesis of various inflammatory diseases (Mangan et al., 2018). Nevertheless, the role of the NLRP3 inflammasome in CNS trauma still remains complex and controversial. Here, we focus on the molecular mechanisms of NLRP3 inflammasome in the pyroptosis signaling pathway, including its prime, activation, and post-translational modifications. Moreover, the dynamic expression pattern, and roles of the NLRP3 inflammasome in CNS trauma are summarized. The pharmacological applications of NLRP3 inflammasome inhibitors in CNS trauma are also discussed.

## 2 Pyroptosis and Inflammasomes

Pyroptosis is a novel inflammatory type of programmed cell death (PCD) that is linked to the activation of inflammasomes (Ousingsawat et al., 2019). Pyroptosis was originally described in 1992 by Zychlinsky et al. (1992), who found that a cell death form of lysis way of infected macrophages caused by *Shigella flexneri*. It was formerly believed to be apoptosis because it shares some features with apoptosis, including DNA fragmentation, nuclear pyknosis (chromatin condensation), and caspase dependence (Ye et al., 2019). However, it is not identical to apoptosis, especially in terms of its morphological features. Subsequent research detected macrophages infected by various pathogens such as *Salmonella typhimurium*, *Listeria monocytogenes*, and *Pseudomonas aeruginosa*. Caspase-1-dependent cell death releases a large amount of pro-inflammatory factors (Jesenberger et al., 2000; Breitbach et al., 2009; Wu et al., 2010). Caspase-1 plays a significant role in the maturation of pro-inflammatory cytokines. Once triggered, caspase-1 potentially catalyzes the conversion of inactive precursors interleukin-1β (IL-1β) and interleukin-18 (IL-18) into mature bioactive forms, thus magnifying innate and adaptive immune responses (Ippagunta et al., 2011). In 2001, Cookson and Brennan (2001) first used the term “pyroptosis” to define this novel type of caspase-1-dependent regulated cell death, which was used to distinguish it from apoptosis. Pyroptosis is correlated with the progression of various human diseases, including cancer, neurodegenerative disease and cardiovascular disease (Ma et al., 2018). Furthermore, the role of pyroptosis in the development of CNS post-traumatic injury has garnered significant interest in research.

**FIGURE 1:**
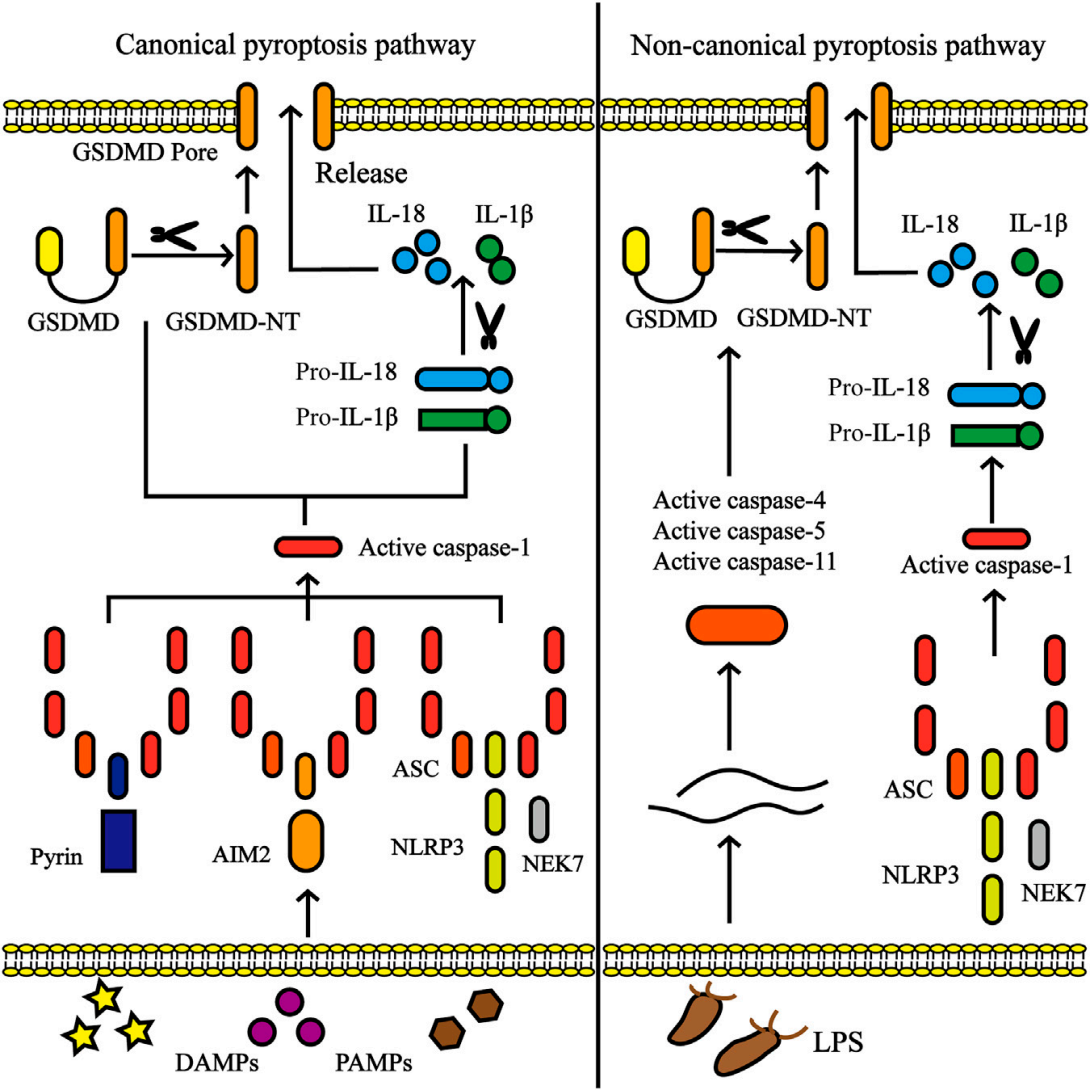
Canonical pyroptosis pathway and non-canonical pyroptosis pathway. In the canonical pyroptosis pathway, activation of inflammasome (including NLRP3, AIM2 or pyrin) occurs *via* a multitude of pathogen-associated molecular patterns (PAMPs) or damage-associated molecular patterns (DAMPs). Once activated, NLRP3 oligomerizes through homotypic interactions of their NACHT domains. The oligomerized NLRP3 recruits ASC via homotypic PYD-PYD interactions and subsequently recruits caspase-1 through CARD-CARD interactions. The activated caspase-1 involves the cleavage of pro-IL-1β/18 into IL-1β/18 and Gasdermin D (GSDMD) into N-terminal fragment of GSDMD (GSDMD-NT), which forms the pores in the plasma membrane, accompanied by the secretion of IL-1β/18. In non-canonical pyroptosis pathway, caspase-4/5/8/11 are activated by intracellular lipopolysaccharide (LPS) derived by bacteria, leading to pyroptosis by the cleavage of GSDMD.

Pyroptosis involves cell swelling, pore formation in the plasma membrane, membrane rupture, significant inflammation, and release of a large amount of cytosolic contents (Shi et al., 2015). There are two pyroptosis signaling pathways: the canonical (caspase-1 dependent) pathway and the non-canonical (caspase-1 independent) pathway (Figure 1). The pyroptosis pathway is inseparable from inflammasomes. The assembly of the inflammasome is a feature of the inflammatory cascade, which is a multiprotein complex expressed in the cytoplasm of the cell and is composed of pattern recognition receptor (PRR) activation (Ferreri et al., 2010). PRRs are involved in recognizing specific patterns of microorganisms and are key to detecting pathogen invasion. PRRs are further classified into five families: Toll-like receptors (TLRs), NOD-like receptors (NLRs), C-type lectin receptors (CTLs), AIM2-like receptors (ALRs), and retinoic acid-inducible gene (RIG)-I-like receptors (RLRs), among which the main ones closely tied with pyroptosis are TLRs, NLRs, and ALRs (Lamkanfi and Dixit, 2014; Marongiu et al., 2019). PRRs interact with certain pathogen-associated molecular patterns (PAMPs) from microorganisms or nonpathogen-related damage-associated molecular patterns (DAMPs) released by host cells under damaged conditions to trigger the innate immune responses (Reed et al., 2013).

**FIGURE 2:**
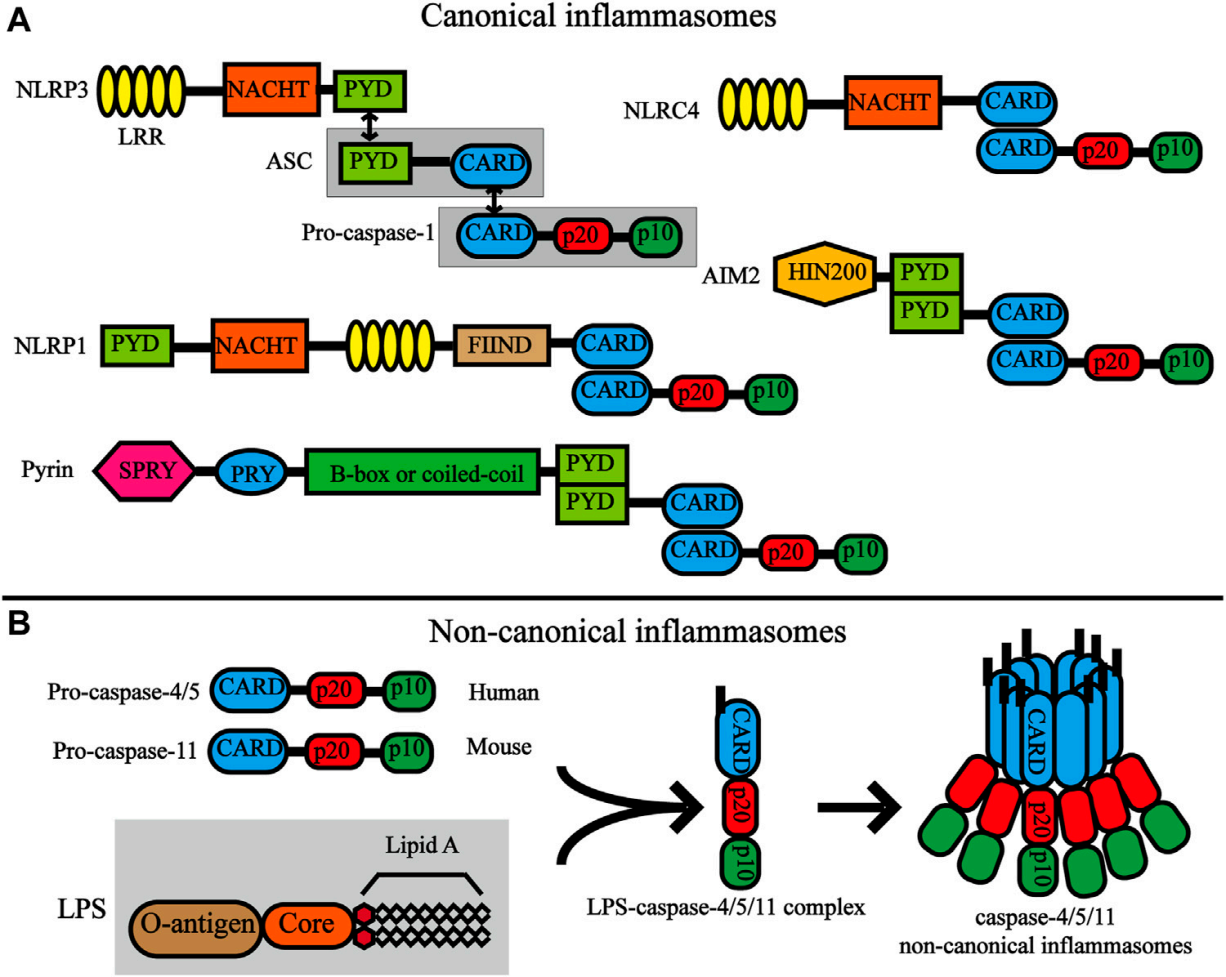
Structures of canonical inflammasomes and non-canonical inflammasomes. **(A)** Domain organization of canonical inflammasomes. The nucleotide-binding oligomerization domain (NOD)-like receptors (NLRs) have common domains, which include the C -terminal leucine-rich repeat (LRR), NACHT, and the N-terminal effector domains. The black bidirectional arrows represent the interaction between homotype PYD/PYD and CARD/CARD. Double-stranded DNA (dsDNA) sensors absent in melanoma 2 (AIM2) are composed of a C-terminal dsDNA-binding HIN200 domain and an N-terminal PYD. The pyrin protein contains an amino-terminal PYD, a B-box, a coiled-coil, a Spla and Ryanodine receptor (SPRY) domain and a PRY domain. **(B)** Structures and activation of non-canonical inflammasomes. Pro-caspase-11 (mouse) and pro-caspase-4/5 (human) are composed of the same structure, namely CARD, P20 and P10. LPS directly interacts with the CARD motif of pro-caspase-11 or pro-caspase-4/5 through the lipid A moiety of LPS. Activation of caspase-4/5/11 non-canonical inflammasome by oligomerization of the LPS-caspase-4/5/11 complex.

Inflammasomes are also divided into canonical and non-canonical inflammasomes (Figure 2). Canonical inflammasomes include double-stranded DNA (dsDNA) sensors absent in melanoma 2 (AIM2), pyrin inflammasomem, and NLRP subfamily (NLRP1, NLRP3, and NLRC4) (He X.-m. et al., 2020). Non-canonical inflammasomes include mouse caspase-11 and human caspase-4 and caspase-5, and are highly homologous (Yi, 2020). Among the numerous inflammasome complexes, the NLRP3 inflammasome is the most closely related to pyroptosis and is the best-characterized inflammasome. The classic NLRP3 inflammasome consists of three components, namely the NLRP3 receptor, adaptor protein apoptotic speck protein (ASC), and effector caspase-1, of which NLRP3 receptor is the key component and is composed of an N-terminal pyrin domain (PYD), a central nucleotide-binding and oligomerization domain referred to as NACHT and a C-terminal LRR domain. When activated, NLRP3 oligomerizes through homotypic interactions of their NACHT domains. Oligomerized NLRP3 recruits ASC via homotypic PYD-PYD interactions and subsequently recruits caspase-1 through CARD-CARD interactions (Lu et al., 2014; Schmidt et al., 2016; Boucher et al., 2018). NLRP3 inflammasomes are mainly expressed in immune cells (monocytes, dendritic cells, neutrophils, and lymphocytes), epithelial cells, and osteoblasts (Zhong et al., 2013). Moreover, recent studies have shown that NLRP3 inflammasomes are expressed in the microglia and neurons in the brain and spinal cord as well (Chen et al., 2019b; Chen et al., 2019c).

Several reports have shown that NIMA-related kinase 7 (NEK7), a mitotic serine/threonine kinase, is an essential component of NLRP3 inflammasome activation through interaction with NLRP3 (Huang et al., 2018). The two domains of NEK7, the N-lobe and the C-lobe, interact directly with NLRP3, and NEK7 oligomerizes NLRP3 to form a functional complex, which is closely related to ASC oligomerization and ASC speck formation (Chen X. et al., 2019; Shi et al., 2020). Interestingly, K^+^ efflux is a driver event in upstreaming of NEK7-mediated NLRP3 activation (Chen et al., 2019b). Once NEK7 binds to NLRP3, it triggers a series of caspase cascade and induces pyroptosis. Therefore, NEK7, as an upstream regulator of NLRP3 inflammasome activation, may be a potential therapeutic target.

## 3 Roles of NLRP3 Inflammasome in Pyroptosis

Although the underlying activation mechanism of the NLRP3 inflammasome is still not clearly elucidated, two signal activation mechanisms are currently generally accepted. Two signals are required for the NLRP3 inflammasome to function: the priming signal (signal 1) and activation signal (signal 2).

### 3.1 Priming of NLRP3 Inflammasome

The priming signal is a prerequisite for the activation signal, and its main function is to up-regulate the inflammatory component and make it in a state of inactivation but capable of responding to the signal. The priming signal refers to the activation of the NF-κB signaling pathway through PRRs receptors, such as TLRs, NLRs, or cytokine receptors that activate the transcription factor NF-κB, including tumor necrosis factor (TNF) and interleukin-1β (IL-1β) (Bauernfeind et al., 2009). Subsequently, activated NF-κB upregulated the expression of inflammation-related genes *NLRP3*, *pro-IL-1β*, and *pro-IL-18*, which are considered to play a transcriptional upregulatory role (Bauernfeind et al., 2009; Pecorelli et al., 2020). Strikingly, although NF-κB is upstream of NLRP3, overexpression of NLRP3, in turn, activates the NF-κB signaling pathway (Peng et al., 2020). In addition, it has been reported that the initiation of the NLRP3 inflammasome is also affected by FAS-associated death domain protein (FADD) and non-apoptotic caspase-8 (Allam et al., 2014; Gurung et al., 2014). Caspase-8 deficiency inhibits the activation of caspase-1 and caspase-11 by NLPR3 (Gurung et al., 2014). Caspase-8 not only promotes NLRP3 inflammasome activation and IL-1/IL-18 release but also separates gasdermin D (GSDMD) into the functional N-terminal fragment of GSDMD (GSDMD-NT) domain that triggers pyroptosis (Orning et al., 2018; Sarhan et al., 2018). Recent studies have reported that cellular FLICE-inhibitory protein (c-FLIP) contains a caspase-like domain similar to caspase-8, which can also cleave GSDMD to release the GSDMD-NT (Huang et al., 2021). FADD is responsible for recruiting pro-caspase-8 and converting it into active caspase-8, leading to the initiation of the caspase cascade (Martinet et al., 2019). Among the TLR/IL-1R signaling pathway, TRAF6 mediates non-transcriptional priming of the NLRP3 inflammasome in the presence of ubiquitin E3 ligase activity (Xing et al., 2017). Moreover, as signal molecules downstream of TLRs and MyD88, IL-1 receptor-associated kinase 1 (IRAK-1) and IRAK-4 play an important role in the rapid transcription independent of NLRP3 priming (Fernandes-Alnemri et al., 2013; Lin et al., 2014). At the priming stage, BRCA1/BRCA2-containing complex subunit 3 (BRCC3), a deubiquitinating enzyme, triggers the deubiquitination of the LRR region of NLRP3, promoting the activation of the NLRP3 inflammasome (Juliana et al., 2012; Py et al., 2013). Additionally, JNK1-mediated NLRP3 phosphorylation at S194 plays a key role in NLRP3 deubiquitination (Song et al., 2017). In summary, these different intracellular signaling molecules are involved in NLRP3 inflammasome priming at both the transcriptional and post-translational levels.

### 3.2 Activating of NLRP3 Inflammasome

**FIGURE 3:**
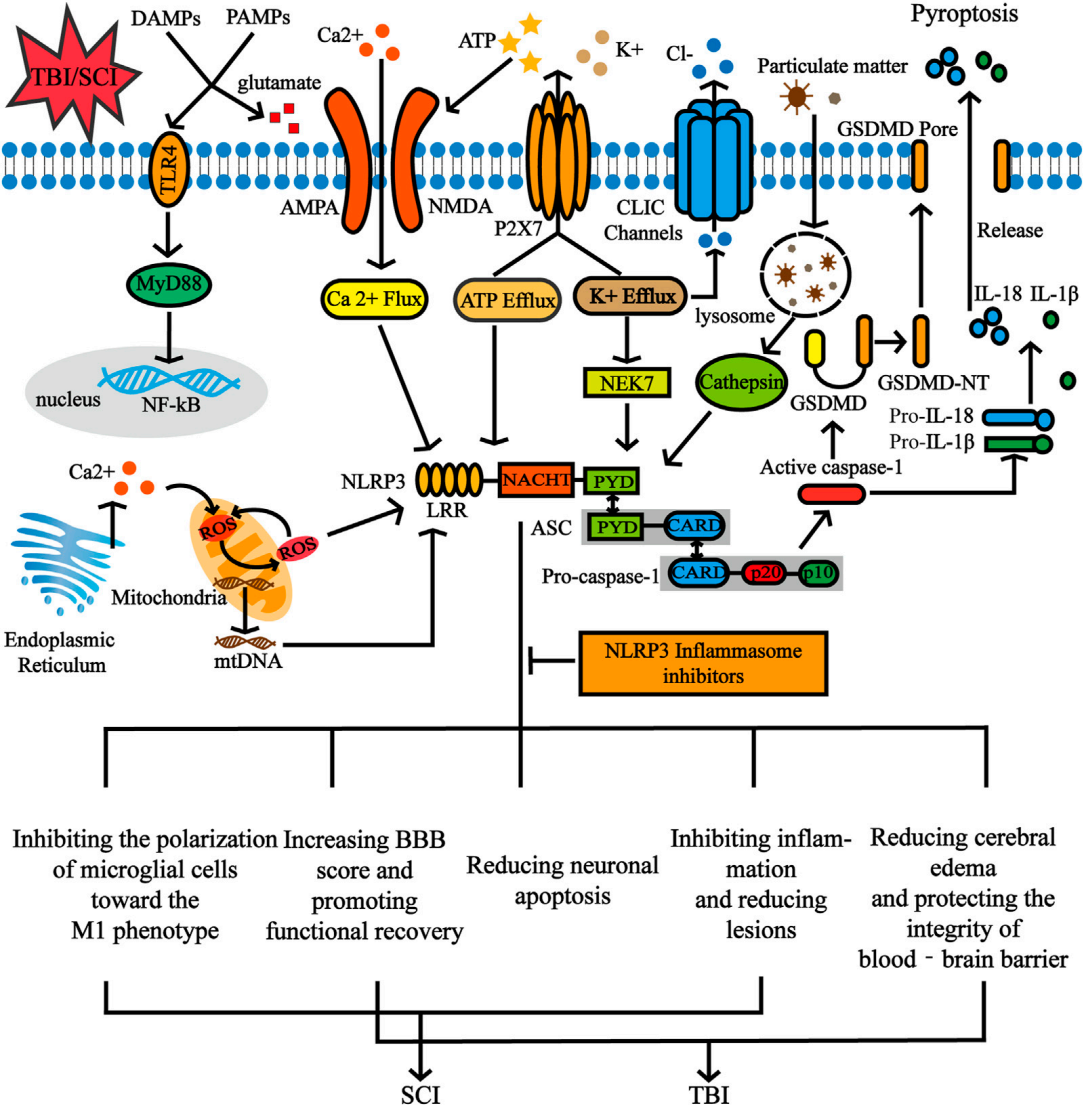
The activation signal of NLRP3 inflammasome and the functional role of NLRP3 inflammasome inhibition in CNS post-traumatic injury. NLRP3 inflammasomes can be activated by various stimuli including ATP and particulate matter. A series of molecular and cellular events, including ionic flux, overproduction of reactive oxygen species (ROS), mitochondrial dysfunction and lysosomal damage have also been demonstrated to trigger the NLRP3. After SCI and TBI, the release of DAMPs and PAMPs is accompanied by an increase in extracellular glutamate levels, which further leads to excitotoxic neuron damage through amino 3 hydroxy-5 methyl-4 isoxazolepropionic acid (AMPA) and N methyl-d aspartate (NMDA) receptors. Furthermore, glutamate-mediated Ca2+ influx occurs through NMDA and AMPA receptors. ATP activates the P2X7 receptor channel and releases large amounts of ATP through this channel. In addition, stimulating the P2X7 receptor results in a decrease in intracellular K+. Inhibition of pyroptosis by NLRP3 inflammasome inhibitors affects biological processes of inflammation, proliferation, apoptosis, etc.

After the priming signal is completed, the activation signal mainly promotes the assembly and activation of NLRP3 inflammasomes and is mediated by related PAMPs or by DAMPs. NLRP3 is known to be activated by a wide variety of stimuli, including ATP, monosodium urate (MSU) crystals, nigericin, pathogen-associated RNA (Mariathasan et al., 2006; Martinon et al., 2006; Halle et al., 2008; Mao et al., 2013). Moreover, a series of molecular and cellular events, including ionic flux, overproduction of reactive oxygen species (ROS), mitochondrial dysfunction, and lysosomal damage have been demonstrated to trigger NLRP3 (Kim et al., 2021). There are certain associations between these molecular and cellular events, and although increasing studies have demonstrated their respective mechanisms, there are still some contradictions (Figure 3).

#### 3.2.1 Ionic Flux

Many studies have demonstrated that changes in intracellular ion flux are the main pathways for NLRP3 inflammasome activation. Ionic flux generally includes potassium, calcium, chloride, sodium, manganese, magnesium, iron and zinc ions (Li et al., 2021). K^+^ efflux is proposed as a common ionic event for multiple-stimuli-induced NLRP3 activation (Seo et al., 2015). It was found that reduction of the intracellular K^+^ concentration can activate the NLRP3 inflammasome signaling and mediate the maturation of IL-1 β in response to nigericin, a K^+^/H^+^ ionophore, and P2X purinoceptor 7 (P2X7) receptor, which binds to ATP (Perregaux and Gabel, 1994; Walev et al., 1995; Surprenant et al., 1996). P2X7 may induce the influx of cations and generate a driving force for K^+^ efflux (Kopp et al., 2019). Importantly, the high concentration of extracellular K^+^ hinders the activation of NLRP3 inflammasomes but does not prevent the activation of NLRC4 or AIM2 inflammasomes (Petrilli et al., 2007; Munoz-Planillo et al., 2013). It is worth noting that as a necessary step for assembling the NLRP3 inflammasome, the binding of NEK7 and NLRP3 requires K^+^ efflux (Chen et al., 2019b). This suggests that K^+^ efflux is an upstream event of NLRP3 inflammasome activation and that K^+^ directly regulates NLRP3. In addition, K^+^ efflux may promote NLRP3 activation by inducing mitochondrial dysfunction and mitochondrial ROS (mtROS) production (Tang et al., 2017). It has recently been reported that NLRP3 inflammasome activation is independent of K^+^ efflux, but the specific mechanism remains unclear and needs further research in the future (Gross et al., 2016; Sanman et al., 2016; Wolf et al., 2016).

Intracellular Ca^2+^ mobilization is also considered to be a fundamental upstream event of NLRP3 inflammasome activation, but its role remains controversial (Murakami et al., 2012). On the one hand, Lee et al. reported that calcium-sensitive receptor (CaSR) induces Ca^2+^ release from endoplasmic reticulum (ER) stores, and Ca^2+^ promotes ASC–NLRP3 association in cell-free lysates of LPS–primed bone marrow-derived macrophage (Lee et al., 2012). Besides CaSR, intracellular Ca^2+^ can also be increased by ligand-gated calcium channels and voltage-dependent calcium channels (VDCCs), ER/sarcoplasmic reticulum (SR) Ca^2+^ stores, or the lysosome (Weber and Schilling, 2014). In addition, increased intracellular Ca^2+^ concentration damages mitochondria, leading to mtDNA production, mitochondrial lipid cardiolipin externalization, and mtROS release, activating NLRP3 inflammasome (Shimada et al., 2012; Iyer et al., 2013; Horng, 2014). Interestingly, this effect is mutual. The overproduction of mtROS not only leads to an increase in intracellular Ca^2+^ but also causes a vicious cycle of ROS, which further increases ROS and mitochondrial damage (Shimokawa et al., 2016). On the other hand, an earlier study showed that regardless of whether the cytosolic Ca^2+^ increases at the same time, K^+^ efflux can effectively activate the NLRP3 inflammasome (Katsnelson et al., 2015). Another study found that NLRP3 activation is independent of Ca^2+^ flux, or that NLRP3 activation is an upstream event of Ca^2+^ flux (Katsnelson et al., 2015).

#### 3.2.2 ROS and Mitochondrial Dysfunction

Increased levels of ROS, a major metabolite of oxidative stress, trigger the activation of the NLPR3 inflammasome and the release and maturation of IL-1β (Vigano et al., 2015). Various sources of ROS in cells are known, and the main ones that have been deeply studied are the mitochondria and NADPH oxidase system (Mantel et al., 2012). Mitochondria play a key role in a variety of cellular functions, including ROS production, ATP production, and calcium homeostasis regulation. Mitochondrial damage can be induced by a variety of factors, such as LPS, ATP, and inhibitors of mitophagy, followed by mtROS production and mitochondrial DNA (mtDNA) release into the cytoplasm (Zhou et al., 2011; Kim et al., 2016). Mitophagy can clear damaged mitochondria and control the level of mtROS; thus, it is an important regulatory means to prevent further activation of NLRP3 (Sciarretta et al., 2012). The release of mtDNA in the cytoplasm is attributed to the opening of the mitochondrial permeability transition (MPT) pore, and the opening of the MPT pore is the result of the direct oxidation of MPT components by a large number of ROS (Nakahira et al., 2011; Yu et al., 2019a). Shimada et al. showed that the oxidized form of mtDNA activates NLRP3 more vigorously, whereas the non-oxidized form of mtDNA is primarily inclined to activate AIM2 inflammasomes (Shimada et al., 2012).

More recently, reports also support that newly synthesized oxidative mtDNA enhances the activation of the NLRP3 inflammasome (Zhong et al., 2018). In addition, oxidation of mtDNA occurrs in severe fever with thrombocytopenia syndrome virus (SFTSV)-infected macrophages even without new mtDNA synthesis, suggesting that oxidative mtDNA is a more critical upstream event for NLRP3 inflammatory activation (Li S. et al., 2020). A recent study indicated that ELABELA (ELA) overexpression inhibits NADPH oxidase activity, which produces ROS, thereby inhibiting the activation of NLRP3 inflammasomes (Chen Z. et al., 2020). NADPH oxidase 2 (NOX2) is involved in oxidative stress in the pathological process of TBI, and the knockout of NOX2 reduces the activation of the NLRP3 inflammasome and the expression of inflammatory components (Ma et al., 2017). Moreover, NADPH oxidase four induces the activation of NLRP3 through carnitine palmitoyltransferase 1(CPT1)-mediated fatty acid oxidation (FAO) (Batista-Gonzalez et al., 2019).

#### 3.2.3 Lysosomal Damage

Among several stressors that trigger the activation of NLRP3 inflammasomes, lysosomal rupture is an important factor, but the crosstalk between lysosomal rupture and NLRP3 inflammasome is not fully understood. Lysosome rupture is mainly caused by the phagocytosis of particulate matter, including silica, aluminum hydroxide, L-leucyl-L-leucine methyl ester and MSU (Munoz-Planillo et al., 2013). Particulate matter in the air induces inflammation through classical or non-classical pyroptosis pathways. Previous studies have documented that airborne particulate matter (PM2.5) enters cells through endocytosis and activates NLRP3 inflammasomes to cause lung inflammation and fibrosis (Zheng R. et al., 2018). Additionally, nanoparticles produced by combustion promote the release of IL-18 and IL-33 through non-classical caspase-4-dependent pathways and are not associated with NLRP3 (De Falco et al., 2017). Lysosomal rupture releases cathepsin, especially cathepsin B (CTSB), which is involved in the activation of the NLRP3 inflammasome via interaction at the endoplasmic reticulum level (Chevriaux et al., 2020).

Studies have shown that CTSB is an upstream activator of the NLRP3 inflammasome, promoting the release of IL-1β, and this effect can be inhibited by the CTSB inhibitor CA-074-Me5 (McHugh et al., 2019; Demirel et al., 2020). Additionally, CTSB inhibits autophagy by regulating the kinase ULK1 activity, and autophagy negatively regulates the activation of NLRP3 inflammasomes (Qi et al., 2016; Zhao et al., 2021). Similarly, recent evidence has illustrated that the use of autophagy inhibitors reversed the effect of betulinic acid, which reduces the activation of pyroptosis after SCI(Wu et al., 2021). However, evidence has shown that IL-1β release is not affected even in the absence of CTSB, thus casting doubt on the role of CTSB in NLRP3 inflammasome activation (Halle et al., 2008; Bauer et al., 2010; Hari et al., 2014).

## 4 Dynamic Activation Pattern, and Roles of NLRP3 Inflammasome in CNS Trauma

**FIGURE 4:**
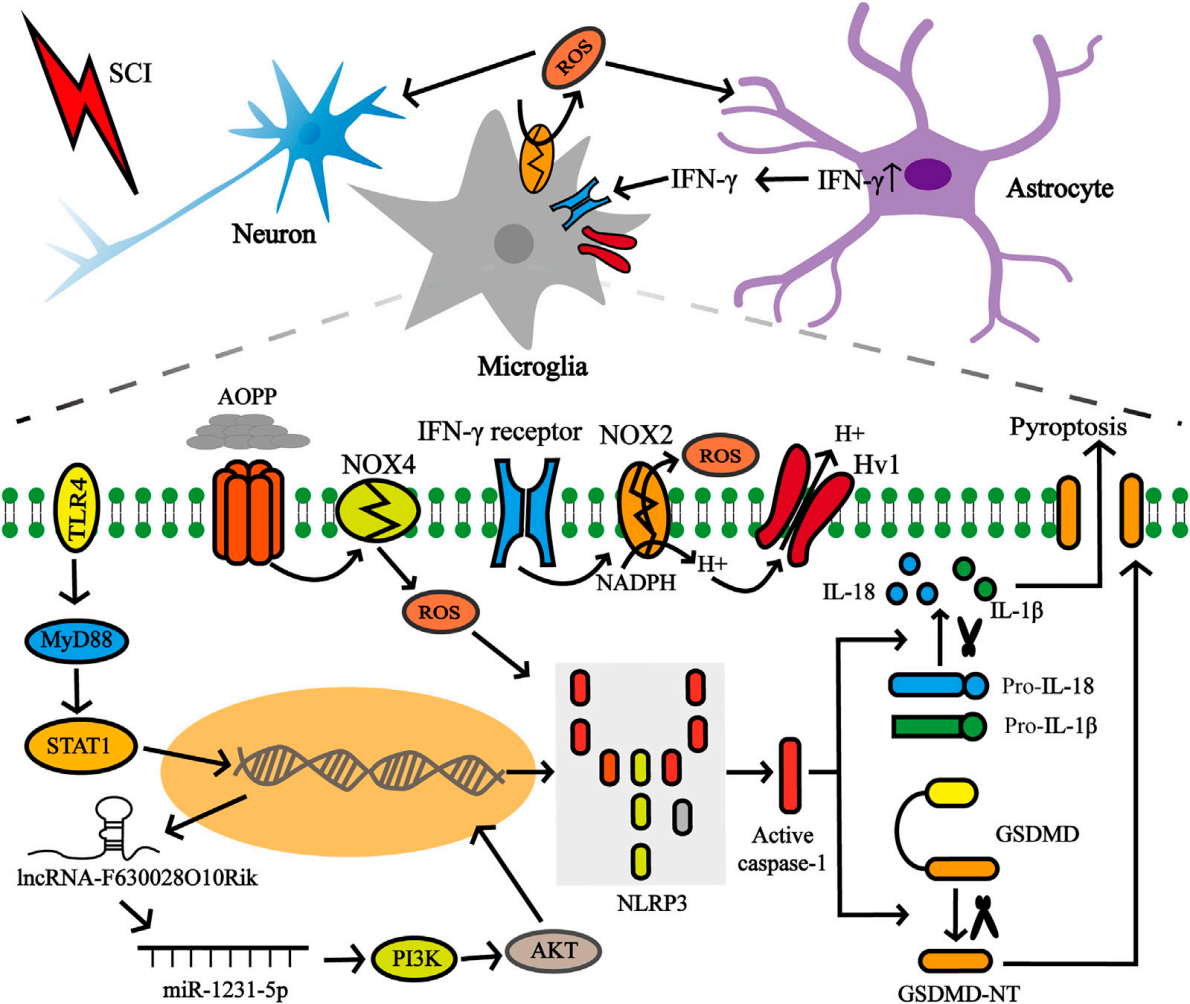
NLRP3 inflammasome-dependent pyroptosis in CNS post-traumatic injury. **(A)** A variety of cell types are involved in CNS pyroptosis, including microglia, neurons, and astrocytes, of which microglia are the most dominant. **(B)** The dynamic activation pattern of NLRP3 Inflammasome.

Previous studies have demonstrated that NLRP3 is involved in the assembly of inflammasomes in CNS diseases and exists mainly in microglia, which are the earliest responders of CNS pathological injury (Saijo et al., 2013; Mamik and Power, 2017; Chen Y. et al., 2020). Furthermore, other researchers discovered the presence of NLRP3 inflammasome in neurons and astrocytes (Qian et al., 2017; Chen Y. et al., 2020). Since NLRP3 is the most abundant molecule among all types of inflammasomes, especially in the CNS, the dynamic activation pattern and roles of the NLRP3 inflammasome in CNS trauma was demonstrated by *in vivo* and *in vitro* experiments (Figure 4).

### 4.1 NLRP3 Inflammasome in SCI

In the early stage, after SCI, NLRP3 expression levels were remarkably upregulated in the spinal cord contusion lesion rat model at 72 h post-injury and in LPS + ATP-induced murine BV2 microglial cells relative to the sham model group (Liu et al., 2021). Moreover, increased protein expression of NLRP3 was also found in T13 dorsal root avulsion model rats (Ellis et al., 2016). Consistently, in the SCI mouse model, quantitative analysis of western blot results revealed that the NLRP3 was significantly enhanced after injury. In addition to NLRP3, an immunofluorescence experiment showed an increased density of GSDMD and caspase-1 in the spinal cord lesions, suggesting that SCI activates the NLRP3 inflammasome with the consequent increase in the pyroptosis-related markers (Wu et al., 2021).

Consistent with previous results, Zheng et al. showed that mRNA levels of NLRP3 and NLRP1 began to increase at 24 h in a rat model of SCI, peaked on day 3, and gradually decreased within 7 days. Moreover, the protein expression of NLRP1 and NLRP3 was elevated within 6 h in primary neurons after injury. Throughout the research process, NLRP3 expression changed more significantly than NLRP1 (Zheng et al., 2019). Zendedel et al. reported that NLRP1 mRNA levels immediately rose within 6 h post-SCI, but this trend was completely reversed. Before 3 days, NLRP3 was always on the rise and reached its peak, whereas the NLRP1b gradually declined and reached a baseline value. After 72 h, NLRP3 began to decline gradually until day 7, while NLRP1b remained at its baseline level (Zendedel et al., 2016). In a recent study, they also found significant increases in ASC, NLRP3, NLRP1b, and NLRC4 mRNA expression levels after SCI in rats, as well as ASC and NLRP3 protein levels. However, there was a slight decrease in the protein expression of NLRP1b, and notably, no detectable expression of NLRC4 (Zendedel et al., 2018). In addition, Jiao et al. reported that NLRP3 expression levels gradually increased after injury, peaked on the second day, and then gradually decreased in SCI mice (Jiao et al., 2020). Clinically, the authors of a study reported that the level of NLRP3 expression in the peripheral blood samples of SCI patients was significantly higher than that in normal patients and was positively related to the severity of the injury. In particular, they also found that compared with the NLRP3/GSDMD low expression group, the high level group of the NLRP3/GSDMD had a higher neck disability index (NDI) and a lower Japanese Orthopedic Association (JOA) score (Xu et al., 2021).

**FIGURE 5:**
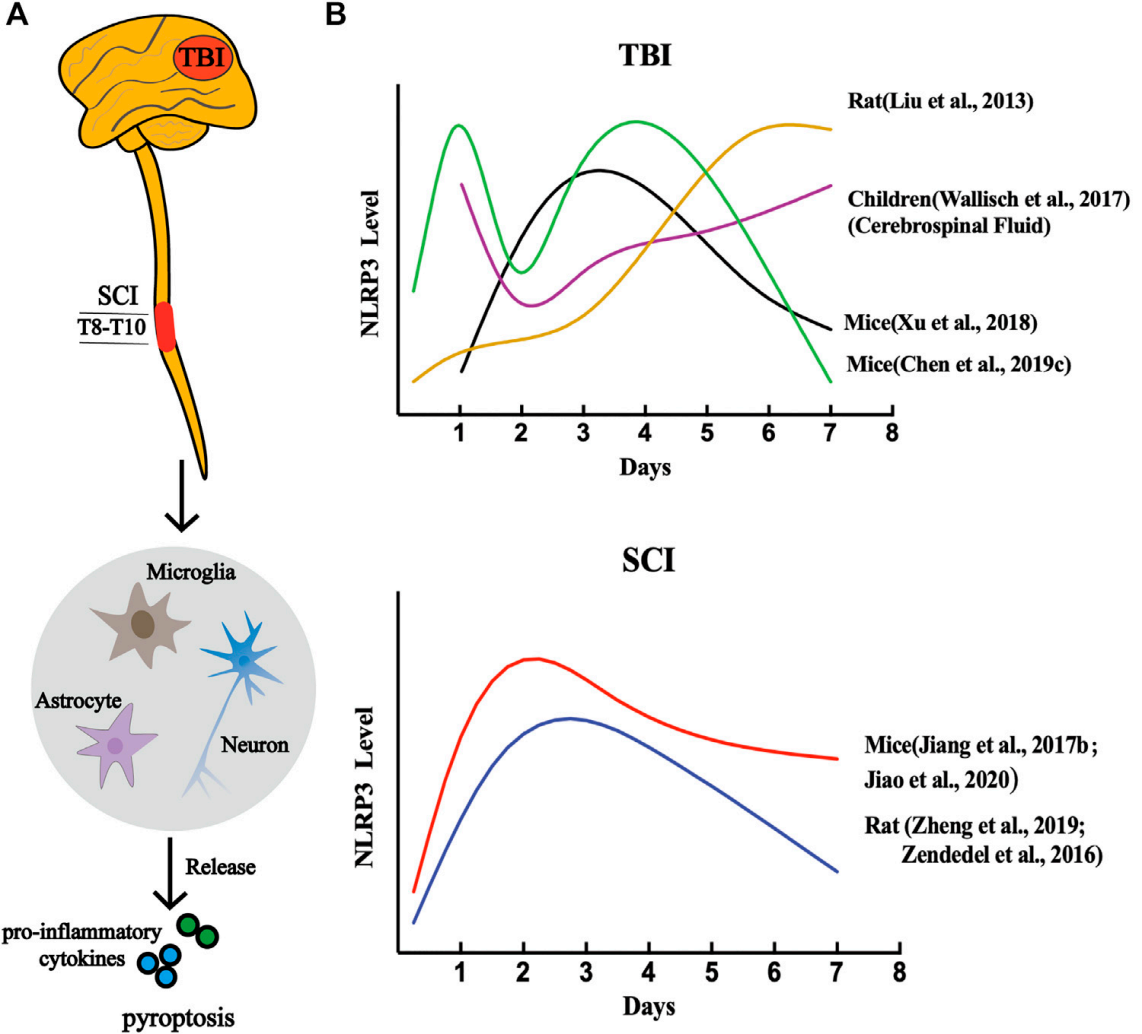
Factors affecting NLRP3 in microglia after SCI. SCI injury signals were transmitted to microglia via TLR4/MyD88/STAT1 network, which subsequently activated the lncRNA-F630028O10Rik/miR-1231-5p pathway, and subsequently triggered pyroptosis. In addition, the increase of Hv1 in microglia post-injury contributes to ROS production. ROS production promotes IFN-γ release from astrocytes, and IFN-γ in turn strengthens microglia activation and microglia-astrocyte interaction. ROS production is also closely related to AOPP.

Additional studies have compared the dynamic expression pattern of the pyroptosis-related key gene *NLRP3* after SCI (Figure 5). Xu et al. showed that SCI injury signals are transmitted to microglia via TLR4/MyD88, which phosphorylate STAT1 and trigger the lncRNA-F630028O10Rik/miR-1231-5p pathway, and finally enhance pyroptosis via PI3K/AKT. In the injured spinal cord segment of TLR4 −/−mice, the protein and mRNA expression levels of NLRP3 were sharply reduced 3 days post-injury, as well as in BV2 microglial cells treated with the TLR4 inhibitor TAK242 compared to the wild-type (WT) sham-operated group (Xu et al., 2020). As mentioned earlier, the increase in ROS levels is a common event that activates NLRP3 inflammasomes and is regulated by the voltage-gated proton channel (Hv1) (Patel et al., 2019). The latest research shows that Hv1 in spinal microglia increases significantly after spinal nerve transection and contributes to the production of ROS. ROS act on neurons and astrocytes, causing IFN-γ release, which in turn further activates microglia and microglia-astrocyte interactions (Peng et al., 2021). The number of NLRP3-positive neurons in SCI mice lacking Hv1 was always significantly lower than that in the WT group, and reached a peak on the third day after the injury, and the number on the seventh day was lower than that on the first day (Li et al., 2020c). Additionally, NADPH oxidase-mediated ROS production was closely associated with advanced oxidation protein products (AOPPs) and presented in a time- and dose-dependent manner (Sun et al., 2016). Liu et al. found that AOPP levels in the plasma, cerebrospinal fluid and spinal cord were elevated after SCI and peaked on day 3, followed by a gradual decline, similar to the trend detected in NLRP3 after SCI. The authors have also demonstrated that compared to BV2 cells incubated with LPS + ATP, the relative band ratio of NLRP3/GAPDH was slightly higher in the AOPPS-MSA group (50 μg/ml), but remarkably enhanced in the AOPPS-MSA group (100 and 200 μg/ml) (Liu Z. et al., 2020).

### 4.2 NLRP3 Inflammasome in TBI

TBI also induces the expression of NLRP3 inflammasome components (NLRP3, ASC, and caspase-1) in the cerebral cortex, cerebrospinal fluid (CSF), and peripheral blood in both humans and rodents. In the peri-contusional cortex 24 h after injury, the protein and mRNA levels of NLRP3, ASC, and caspase-1 were augmented mainly in mice with cold brain injury (Wei et al., 2016). In addition, controlled cortical impact (CCI) induced significant expression of NLRP1, NLRP3, NLRC4, ACS, and caspase-1 in brain microvascular endothelial cells from the injured cerebral cortex of mice at 3 days (Ge et al., 2018). High expression of NLRP3 and caspase-1 was also detected in blast-induced TBI rat models at 12 and 24 h post-injury, and rats were subjected to penetrating ballistic-like brain injury after 4 h (Ma et al., 2016; Lee et al., 2018). More importantly, NLRP3 elevated levels were examined in the peripheral blood of neonatal hypoxic-ischemic encephalopathy patients and the CSF of children with severe TBI(Wallisch et al., 2017; Lv et al., 2020). Histologically, NLRP3−/− mice subjected to an impact-acceleration model of diffuse TBI showed less damage to brain structures compared to WT mice (Irrera et al., 2017). ASC levels in serum and serum-derived extracellular vesicles were markedly higher in TBI patients with lung injury than in patients without complications (Kerr et al., 2019). ASC neutralization markedly reduced caspase-1 activation and cerebral contusion volume after post-TBI (de Rivero Vaccari et al., 2009).

Liu et al. reported that NLRP3 mRNA expression began to increase and reached a peak value at 6 h post-TBI in rats using the modified Feeney’s model. After this peak, NLRP3 expression gradually decreased within 24 h, but then continued to increase until 7 days, and its expression was higher than that at 6 h. Notably, in the same study, NLRP3 protein levels continued to increase significantly over time after injury (Liu et al., 2013). Similarly, CSF concentration of NLRP3 in children with severe TBI instantly rose and peaked at 24 h, then it declined at day 2 but was still higher than that in the control group, and then increased again at day 3. In addition, they also found that young TBI children with TBI <5 years old have higher levels of NLRP3 in the CSF than patients ≥5 years old, indicating a worse poor outcome (Wallisch et al., 2017). A possible explanation for this similar trend is that after TBI, disturbances in cellular ionic homeostasis, including K^+^ efflux and Ca^2+^ influx, and the outflow of ATP from dying cells and traumatic lysis led to the first elevation of NLRP3. The latter increase in NLRP3 may be due to secondary injury, including cellular stress and inflammatory cascade (Liu et al., 2013). Interestingly, unlike previous studies, authors from a study documented that, in the TBI mouse model, NLRP3 mRNA levels gradually increased and peaked at 24 h, then fell gradually until the next day, rose again within 72 h, and finally gradually decreased at day 7 (Chen et al., 2019c). In the peri-contusional cortex, the protein levels of NLRP3, caspase-1, and ASC started to increase at day 1 and peaked at 72 h in mice were subjected to the CCI injury; on day 7, the protein expression was decreased but remained higher than in controls (Xu et al., 2018). Therefore, this suggests that the dynamic expression pattern of NLRP3 is influenced by multiple factors. Moverover, understanding the time-course change of the NLRP3 inflammasome contributed to the determination of the appropriate dose, dose interval, and frequency of drug administration, which may improve the therapeutic effect.

Data from several experiments also suggest that not all inflammasome components increase after injury. Immunoblot analysis results demonstrated that caspase-1, caspase-11, and ASC substantially increased in the rat model 15 min following a moderate parasagittal fluid-percussion injury, but NLRP1 did not change significantly (de Rivero Vaccari et al., 2009). In addition, Liu et al. showed that NLRP3 mRNA levels changed significantly after TBI, except for pro-caspase1 (Chen et al., 2019c). In children with severe TBI, NLRP1 in the CSF was detected in only a small number of patients and was elevated only during the first day and undetectable thereafter (Wallisch et al., 2017). With the activation of NLRP3, highly expressed IL-1β and IL-18 were detected in the CSF of TBI patients and the serum of SCI patients and these cytokines are closely related to the pathogenesis of neuroinflammation (Helmy et al., 2011; Kerr et al., 2018; Jiao et al., 2020). Interestingly, the dynamic expression patterns of not only NLRP3 inflammatory components after CNS injury but also inflammatory cytokines, vary widely in the literature. In most cases, the time course of IL-1β and IL-18 was consistent with the changes in NLRP3, suggesting that NLRP3 inflammasome activation is involved in the inflammatory response after CNS injury.

## 5 NLRP3 Inflammasome Inhibitors in CNS Trauma

The NLRP3 inflammasome, as a central part of the cell pyroptosis process, is extensively involved in CNS injury, making it an attractive therapeutic target. Various promising NLRP3 inhibitors have been reported, although none have been approved for the treatment of SCI and TBI. In this paper, several important NLRP3 inhibitors and their mechanisms have been described. (Table 1).

**TABLE 1:** Specific inhibitory compounds of NLRP3 inflammasome activation.

Agents	Host	Animal model	Pharmacological effects	Specificity	References
MCC950	Primary cortical neurons	SCI	Reducing neuronal apoptosis and promoting functional recovery	NLRP3	Ismael et al. (2018), He et al. (2020a)
	C57BL/6 mice	TBI	Reducing neurological severity score and brain water content, repressing NLRP3, ASC, caspase-1, IL-1β, TNF-a, NF-kB/p65, caspase-3, and PARP after TBI		
CY-09	SD rats	SCI	NACHT ATPase inhibitor; binding to Walker A motif; and inhibiting BzATP	NLRP3	Fan et al. (2020)
Bay 11-7082	C57BL/6 mice	SCI	Reducing neuronal death promoting motor recovery and alleviating secondary injury after SCI	NLRP3	Jayakumar et al. (2014), Jiang et al. (2017b), Zheng et al. (2018a), Jiang et al. (2019)
	CCI rats	TBI	Reducing increased brain water content in rats after fluid percussion injury and alleviating TBI-induced injury	NLRC4	
Tranilast	SD rats	SCI	Inhibiting SCI acute stage inflammation and fibrosis, confining lesions around the cavity	NLRP3	Hanada et al. (2014), Lu et al. (2020)
	KunMing mice	TBI	Down-regulating phosphodiesterase-4 B (PDE4B) expression, inhibiting neuroinflammation and reducing bleeding areas in TBI mice		
Rubesin	C57BL/6 mice	TBI	Inhibiting NLRP3 inflammasome and subsequent secretion of IL1β and IL-18, preventing neuronal apoptosis, reducing cerebral edema, and protecting the integrity of BBB	NLRP3	Yan et al. (2020)
MiR-223	CCI mice	SCI	Reducing the neuropathic pain caused by CCI, decreasing the expression of apoptosis and inflammatory factors, and increasing the proportion of M2 macrophages	NLRP3	Zhang et al. (2020), Zhu et al. (2021)
MiR-423-5p	SD rats	SCI	Inhibiting the polarization of microglial cells toward the M1 phenotype	NLRP3	Cheng et al. (2021)
MiR-34c	C57BL/6 mice	SCI	Attenuating CCI-induced neuropathic pain and decreasing inflammatory factors	NLRP3	Xu et al. (2019)
MiR-193a	C57BL/6 mice	TBI	Suppression of miR-193a significantly reduced post-traumatic neuroinflammation and improved neurofunctional recovery	NLRP3	Si et al. (2020)

### 5.1 MCC950

MCC950, a diarylsulfonylurea-containing compound, is considered to inhibit the processing of IL-1β and is also known as a highly potent inhibitor of NLRP3 (Perregaux et al., 2001). Experiments have been conducted in recent years to explore the potential therapeutic effects of MCC950 and have been demonstrated in several disease models, such as diabetes and Alzheimer’s disease (Dempsey et al., 2017; Ward et al., 2019). In addition, MCC950 inhibited caspase-1 activation and subsequent genotype-related IL-1β increase in a murine model of TBI(Xu et al., 2018). A recent study by Baker et al. demonstrated that MCC950 can reverse caspase-4/5—induced IL-1β production and cell death in cells transfected with LPS (Baker et al., 2015). Therefore, MCC950 specifically targets the NLRP3 inflammasome and inhibits the activation of the canonical and non-canonical NLRP3 inflammasome, thus blocking pyroptotic cell death and IL-1β release in response to NLRP3 stimuli. Correspondingly, Coll et al. discovered that although MCC950 blocked NLRP3-induced ASC oligomerization, it did not suppress the priming phase of NLRP3 activation, as well as NLRC4 and AIM2. They further found that MCC950 also hindered IL-1β secretion but did not affect TNF-α in a dose-dependent manner. More specifically, the inhibitory effect of MCC950 may target upstream signaling pathways of the NLRP3 inflammasome because it does not directly inhibit NLRP3–ASC, NEK7–NLRP3, or NLRP3–NLRP3 interactions (Coll et al., 2015). In a recent study, MCC950 was shown to dose-dependently block nigerinin-induced chloride efflux, which may be evidence that MCC950 inhibits upstream signaling events of NLRP3 activation (Jiang H. et al., 2017). However, other upstream ion signaling events, such as K^+^ efflux and Ca^2+^ flux, were not blocked by MCC950 (Coll et al., 2015). Ismael et al. reported that MCC950 significantly inhibited the cleavage of caspase-3 and poly (ADP-ribose) polymerase (PARP), a proapoptotic marker. In addition, the reduction of caspase-3 was associated with the repression of MCC950-associated NF-kB/p65, which strongly indicated the protective effect of MCC950 on apoptotic cell death in TBI lesions (Ismael et al., 2018). Importantly, Xu et al. found that the neuroprotective effect of MCC950 and its ability to reduce cerebral edema were dependent on the presence of microglia and that the therapeutic window of MCC950 was limited within 6 h post-TBI(Xu et al., 2018). Compared with a single drug treatment, MCC950 combined with rapamycin-induced mitophagy further blocked NLRP3 inflammasome assembly and enhanced the neuroprotection in CCI mice (Chen et al., 2019c). Furthermore, MCC950 significantly reduced neuronal apoptosis, improved neuronal survival and functional recovery, and reduced scar formation in SCI mice (He et al., 2020a; Jiao et al., 2020). Although it will take some time for MCC950 to be switched to clinical agents, it represents an important strategy for targeting or addressing CNS trauma because of its specificity and effectiveness in NLRP3 inhibition, as well as its long-term safety and neuroprotective effects of oral administration (Gordon et al., 2018).

### 5.2 CY-09

There is evidence that the ATPase activity of the NACHT-domain of NLRP3 is critical for NLRP3 oligomerization (Duncan et al., 2007). The ATPase activity of NLRP3 has also received extensive attention as a drug target, with a large number of compounds currently receiving attention. Jiang et al. found that a small-molecule CY-09 competitively binds to the Walker A motif of NLRP3, but not Walker B, although the Walker B motif is also necessary for ATPase activity, thus eliminating NLRP3 binding to ATP and blocking its ATPase activity. Notably, CY-09 inhibition also specifically targets NLRP3 ATPase activity and does not affect purified NLRP1, NLRC4, NOD2, or RIG-I ATPase activity. Similarly, CY-09 did not affect LPS-induced priming and TNF-α production (Jiang H. et al., 2017). Unlike MCC950, CY-09 did not block nigerin-induced chloride efflux but effectively inhibited the production of IL-1β and neutrophil influx induced by MSU injection (Jiang H. et al., 2017). Current studies have shown that CY-09 dose-dependently inhibits caspase-1 activation and IL-1β production, but the inhibition of non-classical NLRP3 inflammasome activation requires further study. In microglia of SCI rats, CY-09 significantly inhibited 3′-O-(4-benzoylbenzoyl) adenosine 5′-triphosphate (BzATP), which regulates the NLRP3 inflammasome by upregulating the P2X7 receptor, a key inflammation switch, and ultimately inhibits the development of neuroinflammation (Fan et al., 2020). Increased GFAP expression was significantly attenuated, and astrocyte activation was inhibited by pretreatment with CY-09, which contributes to the prognosis of SCI and TBI (Shen et al., 2020). CY-09 exhibits good pharmacokinetics and directly targets NLRP3 *in vivo*, thus providing a novel strategy to inhibit NLRP3 inflammasome activation.

### 5.3 Bay 11-7082

BAY 11-7082 is a sulfonic derivative with a variety of pharmacological activities. Similar to CY-09, Juliana et al. reported that BAY 11-7082 blocked NLRP3-induced ASC formation by inhibiting NLRP3 ATPase activity, and its effect was dependent on the vinyl sulfone group. However, Juliana et al. observed that Bay 11-7082 did not inhibit NLRP1 ATPase activity, but could partially inhibit NLRC4 inflammasome activation, although the exact mechanism is still unclear (Juliana et al., 2010). Moreover, BAY 11-7082 inhibited microglia/macrophage activation and neutrophil infiltration, reduced glial proliferation and improved mitochondrial dysfunction, thereby reducing neuronal death and promoting motor recovery in SCI mice (Jiang W. et al., 2017). Notably, BAY 11-7082 contributed to improving secondary lung injury after SCI (Jiang et al., 2019). Additionally, in rats subjected to the CCI injury, Zheng et al. showed that the synergistic effect of the co-administration of Bay-11-7082 and dexmedetomidine improved neuronal activity and inhibited microglial activation, as well as hippocampus tissues inflammation (Zheng B. et al., 2018). BAY 11-7082 is also an NF-κB inhibitor that irreversibly represses TNF-α-induced phosphorylation of IκB-α and subsequent nuclear translocation of NF-κB (Jing et al., 2012). In rats inoculated with breast carcinoma cell Walker-256, Wang et al. found a significant increase in monocyte chemoattractant protein-1 (MCP-1)/chemokine CC motif receptor-2 (CCR2) and NF-κB expression in spinal cord neurons, but this effect was inhibited by treatment with BAY 11-7082 (Wang et al., 2018).

A report from Jayakumar showed that BAY 11-7082 administration significantly inhibited NF-κB activation and swelling of astrocytes, inhibited the viability of Na^+^, K^+^, 2Cl^−^ cotransporter, and reduced the high brain water content in rats after fluid percussion injury (Jayakumar et al., 2014). Interestingly, BAY 11-7082 administration inhibited Snail expression increase, the main inducer of epithelial-mesenchymal transition (Jing et al., 2012). Ripk1 is an important upstream regulator of necroptosis and is significantly upregulated after moderate TBI(Zhang et al., 2017). Ripk1 overexpression promoted the activation of the NF-κB signaling pathway and contributed to the release of pro-inflammatory factors and the inhibition of autophagy-related proteins. BAY 11-7082 treatment significantly eliminated these effects and alleviated the TBI-induced injury (Liu J. et al., 2020). Tests on Bay11-7082 are still mostly limited to animals, although they show limited toxicity (Keller et al., 2006). According to the published literature, Bay11-7082 has shown good therapeutic effects in CNS trauma, and future work needs to transition to clinical trials to test its safety and therapeutic effect.

### 5.4 Tranilast

Tranilast is an analog of the tryptophan metabolite and has been used to treat bronchial asthma since 1982 because of its potent anti-inflammatory properties (Darakhshan and Pour, 2015). A recent report by Huang et al. clarified the mechanism underlying the anti-inflammatory effect of tranilast. Like CY-09 and Bay 11-7082, tranilast is also an inhibitor that targets NLRP3. However, tranilast binds directly to the NACHT domain of NLRP3, inhibiting the interaction and oligomerization of NLRP3-NLRP3 and NLRP3 -ASC, but not NLRP3 -NEK7, thereby blocking the assembly of the NLRP3 inflammasome. In addition, tranilast did not affect NLRP3 ATPase activity and specifically inhibited the NLRP3 inflammasome but did not inhibit other inflammasome (Huang et al., 2018). Futhermore, Hanada et al. found that tranilast inhibited inflammation in the acute phase of SCI and confined lesions around the cavity, reducing the formation of TGF-β-mediated fibrotic scarring. They noted that tranilast is safer to administer orally compared to the risk of embolism associated with intravenous administration (Hanada et al., 2014). Second, the combined delivery of tranilast and methylprednisolone had a greater therapeutic effect than the single delivery of either (Mbori et al., 2016). Compound 5, a novel rolipram-tranilast hybrid, has been shown to have strong neuroprotective potential, significantly inhibiting neuroinflammation, and markedly reducing bleeding areas in TBI mice (Lu et al., 2020). Tranilast has been clinically used in other diseases to demonstrate its safety and efficacy. *In vivo* experiments have demonstrated significant therapeutic and prophylactic effects in SCI and TBI mouse models. To strengthen clinical relevance, further studies are needed to identify the tranilast oral dose, treatment window, and more detailed therapeutic effects and mechanisms.

### 5.5 Rubesin

Rubesin is a natural, biologically active diterpenoid derived from the herb Rabdosia, which has been found to have anti-inflammatory, antioxidant and anti-tumor properties (Yu et al., 2019b). Similar to the specific inhibitors mentioned above, oridonin does not interfere with the upstream signaling pathway of the NLRP3 inflammasome and acts directly on the NLRP3 inflammasome itself. In contrast to tranilast, oridonin blocks NLRP3 inflammasome assembly by blocking the NLRP3-NEK7 interaction, but does not affect NLRP3-NLRP3 and NLRP3-ASC. Mechanistically, oridonin specifically binds to the NACHT domain of NLRP3 and forms covalent bonds with cysteine 279 in the NACHT domain, thereby blocking the interaction between NLRP3 and NEK7. In this study, we also found that oridonin did not affect the activation of AIM2 and NLRC4 inflammasomes and the production of TNF-α (He et al., 2018). Activation of the NLRP3 inflammasome and subsequent secretion of pro-inflammatory cytokines IL-1β and IL-18 were significantly inhibited in ORidonin-treated TBI mice. Moreover, oridonin effectively prevented neuronal apoptosis, reduced cerebral edema, and protected the integrity of BBB, showing a strong neuroprotective function (Yan et al., 2020). Although oridonin has shown good therapeutic effects in rheumatoid arthritis, colitis and other inflammatory diseases, it has been shown to be able to treat NLRP3-driven diseases (Liu et al., 2016; He S.-D. et al., 2020). However, there is still a large gap in the study of neuroinflammation in CNS trauma, which needs to be addressed by further research.

### 5.6 MIR-223

MicroRNAs (miRNAs) are small and endogenous non-coding RNA molecules that down-regulate gene expression at the post-transcriptional level (Miyake et al., 2012). MiR-223 is one of the microRNAs that is known to be associated with inflammation and has attracted increasing attention. Bauernfeind et al. revealed that the conserved sites in the 3’untranslated regions of the NLRP3 transcript were bound to the myeloid-specific microRNA miR-223, thus dampening NLRP3 expression and diminishing IL-1β secretion. Meanwhile, miR-223 antagonists contribute to increased levels of NLRP3 protein. Importantly, miR-223 did not affect activation of the AIM2 inflammasome (Bauernfeind et al., 2012). A previous study revealed that MiR-223 can directly inhibit NLRP3 expression, reduce cerebral edema, and inhibit the secretion of inflammatory factors after intracerebral hemorrhage (Yang et al., 2015). Moreover, miR-223 not only protects the brain against glutamate excitotoxicity but also protects dissociated cortical neurons from degeneration in peripheral blood mononuclear cell-conditioned media (Harraz et al., 2012; Morquette et al., 2019). Studies have shown that miR-223 levels are significantly increased after acute SCI, and inhibition of miR-223 aggravates injury and inflammatory response, while overexpression has the opposite effect. The author and colleagues also found that overexpression of miR-223 in microglia inhibits LPS-induced inflammation (Zhang et al., 2020). In addition to what has been described above, several other miRNAs have been shown to be involved in the activation of NLRP3 inflammasome and have shown efficacy in CNS trauma disease models, including miR-423-5p, miR-34c, and miR-193a (Xu et al., 2019; Si et al., 2020; Cheng et al., 2021). Interestingly, downregulation of circ 0001723 induces *NLRP3* expression by upregulating miR-380-3p (Li et al., 2020b). In addition, LncRNA-Meg3 negatively regulates miR-7a-5p to induce microglial inflammation by regulating NLRP3 (Meng et al., 2021). Thus, it is also possible to target miRNAs by regulating other types of non-coding RNAs, such as circRNAs and lncRNAs, thereby regulating NLRP3.

## 6 Conclusion

The pathophysiological processes in SCI and TBI are complicated, and the identification of novel therapeutic targets is urgently needed. Activation of the NLRP3 inflammasome is a central link in pyroptosis and is involved in the pathological development of various inflammatory diseases, including SCI and TBI. While our understanding of the mechanisms of NLRP3 inflammasome priming, activation, and post-translational modification is improving, therapies targeting NLRP3 are advancing rapidly. However, the current treatment of NLRP3 pathology is still limited to animal experiments and is even more restricted to the canonical pyroptosis pathway. It is also important for other NLR or inflammasome sensors or caspases in the non-canonical pyroptosis pathway. The NLRP3 dynamic expression pattern in SCI and TBI shows that the NLRP3 time course of different models has a similar trend. Drug administration can be determined using the corresponding dose and absorption efficiency at different time points in a certain model, which may improve the therapeutic effect. Additionally, it can be combined with clinically effective drugs, such as methylprednisolone. We can also focus on the role of non-coding RNAs in pyroptosis in TBI and SCI. Recent research has shown that bone marrow mesenchymal stem cell-derived exosomes significantly inhibit pyroptosis to improve SCI, and the potential mechanism is to regulate NOD1-related signaling pathways (Zhou et al., 2022). Despite the increasing exosomes from different sources or those carrying a variety of non-coding RNAs, few studies directly target NLRP3 and there are still large gaps in CNS trauma. In summary, further work is still needed to elucidate the role of NLRP3 inflammasome in CNS injury, but increasing drugs are being developed and novel therapies are quickly becoming a reality.
